# Tropical Enteropathies

**DOI:** 10.1007/s11894-017-0570-0

**Published:** 2017-05-24

**Authors:** John Louis-Auguste, Paul Kelly

**Affiliations:** 1 Blizard Institute, Barts & The London School of Medicine, Queen Mary University of London, Newark Street, London, E1 2AT UK; 2 TROPGAN, University of Zambia School of Medicine, University Teaching Hospital, Lusaka, Zambia

**Keywords:** Tropical enteropathy, Environmental enteropathy, Environmental enteric dysfunction, Tropical sprue, Malnutrition

## Abstract

**Purpose of Review:**

The term ‘tropical enteropathy’ originated in observations in the 1960s that small intestinal morphology and function differed in the tropics from the norms found in temperate climates. It was subsequently shown that this enteropathy is more closely related to environmental conditions than latitude, and it was re-labelled ‘environmental enteropathy’. It is now recognised that environmental enteropathy (also now called environmental enteric dysfunction) has implications for the health and linear growth of children in low- and middle-income countries, and it may underlie poor responses to oral vaccination in these countries. The purpose of this review is to define and clarify this enteropathy despite the confusing terminology it has attracted and to contrast it with other enteropathic states.

**Recent Findings:**

Recent work has begun to demonstrate the nature of the mucosal lesion and the relationship with microbial translocation which is currently thought to link a failure of mucosal barrier function and the cascade of systemic inflammation which inhibits growth. The evidence is still correlative rather than definitive, but derives some additional support from animal models. There are some common features between environmental enteropathy and other enteropathies, but there are important differences also. The mechanism of the link between enteropathy and vaccine failure is not understood, and neither is it clear how the more severe form of enteropathy, which we refer to as malnutrition enteropathy, is driven by nutrient depletion and intestinal infection.

**Summary:**

Tropical enteropathies form a group of disorders which include environmental and nutritional enteropathies. The long-term health implications of these disorders for health in low-income countries are just being explored, but the scale of their effects is very large, with millions of people affected.

## Introduction

### The History of ‘Tropical Enteropathy’

A number of investigations into overt, symptomatic diarrhoea and malabsorption in the tropics in the early 1960s used apparently asymptomatic, healthy and well-nourished adults and children from the same population as control subjects. Unexpectedly, they uniformly identified a high prevalence of abnormal intestinal permeability (as measured by urinary sugar recovery) and/or histological abnormalities (villous blunting, crypt hypertrophy, villous fusion and mucosal inflammation) in these controls [1]. Studies in American soldiers and Peace Corps volunteers stationed in Thailand [2] and in Peace Corps volunteers in modern-day Bangladesh [3] showed that the condition was acquired and that these abnormalities were similar to those observed in the indigenous population [4]. Furthermore, histological examination of foetal and neonatal intestine also showed that these abnormalities were not present during development and only became apparent after 6 months.

These changes were reversible, as demonstrated by prospective assessment of small bowel histology and absorption. Peace Corps volunteers who had lived in India or Pakistan returned to histological and absorptive normality, usually within 2 years after returning to the USA [5]. Furthermore, adult students from endemic areas moving to the USA to study also normalised intestinal structure and function [6], and in the UK, it was noted that there was a relationship between villus morphology and the time from last visit to the tropics [7]. Based on the initial studies which were exclusively conducted in the tropics, the condition was labelled ‘tropical enteropathy’ [8].

### The Adoption of ‘Environmental Enteropathy’

However, an extensive worldwide study clearly demonstrated that the observed abnormalities were not observed in some affluent, tropical populations (such as Singapore and Qatar) [9], and the condition is therefore more correctly termed environmental enteropathy (EE). Brunser, working in Santiago which is not tropical, noted the same enteropathy in children living in insanitary slums [10]. Numerous subsequent studies have confirmed that EE is highly prevalent throughout the developing world irrespective of climate and, in particular, is associated with economic conditions [8, 9, 11]. It is seasonal [12].

Early studies of EE demonstrated significant abnormalities in sugar absorption and permeability assays. These tests provide a safe and non-invasive measurement of intestinal permeability and absorptive function, and as such are suited to studies of enteropathy in children in particular [13–15]. Sugar tests have therefore become the de facto test of choice in studies of enteropathy, though recent studies have started to change this. Studies in adults and children typically show reduced urinary recovery of absorbable sugars such as xylose (indicating impaired absorptive capacity) and increased urinary levels of inabsorbable sugars such as lactulose (indicating increased intestinal permeability). The increased intestinal permeability to small sugars as measured by sugar absorption assays is correlated to significant and pathological abnormalities of intestinal barrier function, for example as measured by plasma LPS levels [13, 16]. In turn, chronic endotoxaemia depresses IGF1 secretion providing a mechanism of growth suppression [17••].

There is also increasing evidence from human studies that EE is associated with impaired absorption of macro- and micronutrients. An early study in Bangladeshi children showed that the xylose malabsorption was correlated with some malabsorption of carbohydrates [15]. Children with EE in rural Malawi have disordered zinc homeostasis, with impaired capacity to resorb endogenous zinc in distal small bowel (studied by measuring faecal excretion of isotopic zinc after intravenous administration [18]) regardless of zinc status, resulting in net loss of zinc [14]. There was a strong positive correlation between L/M ratio and excretion of endogenous zinc [14].

### Definition of Enteropathy

**Fig. 1:**
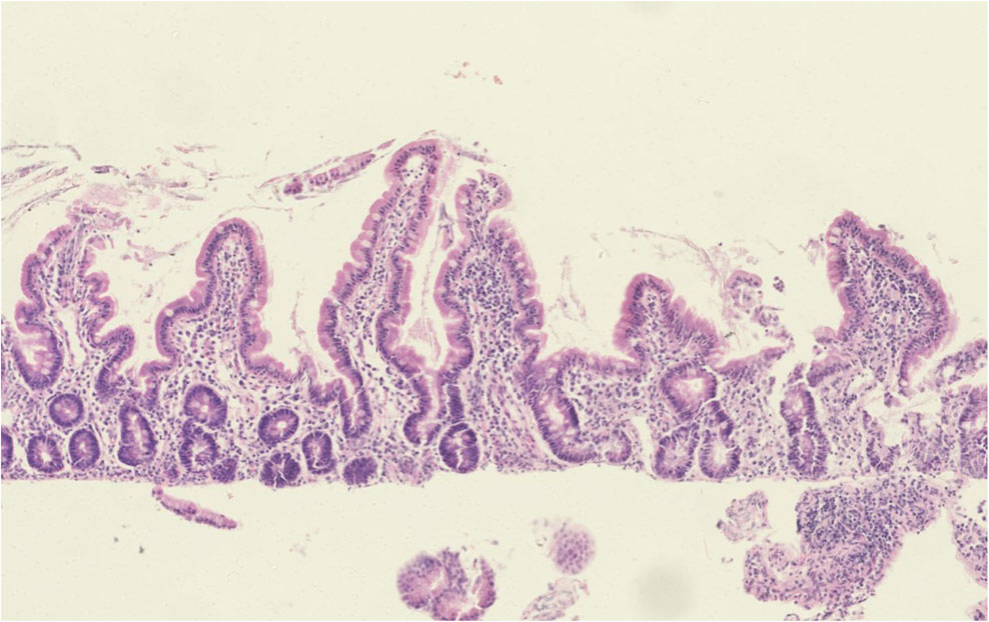
Features of enteropathy in a biopsy from a child with severe acute malnutrition. There is an increased inflammatory cell infiltrate in the lamina propria and villus blunting

**Fig. 2:**
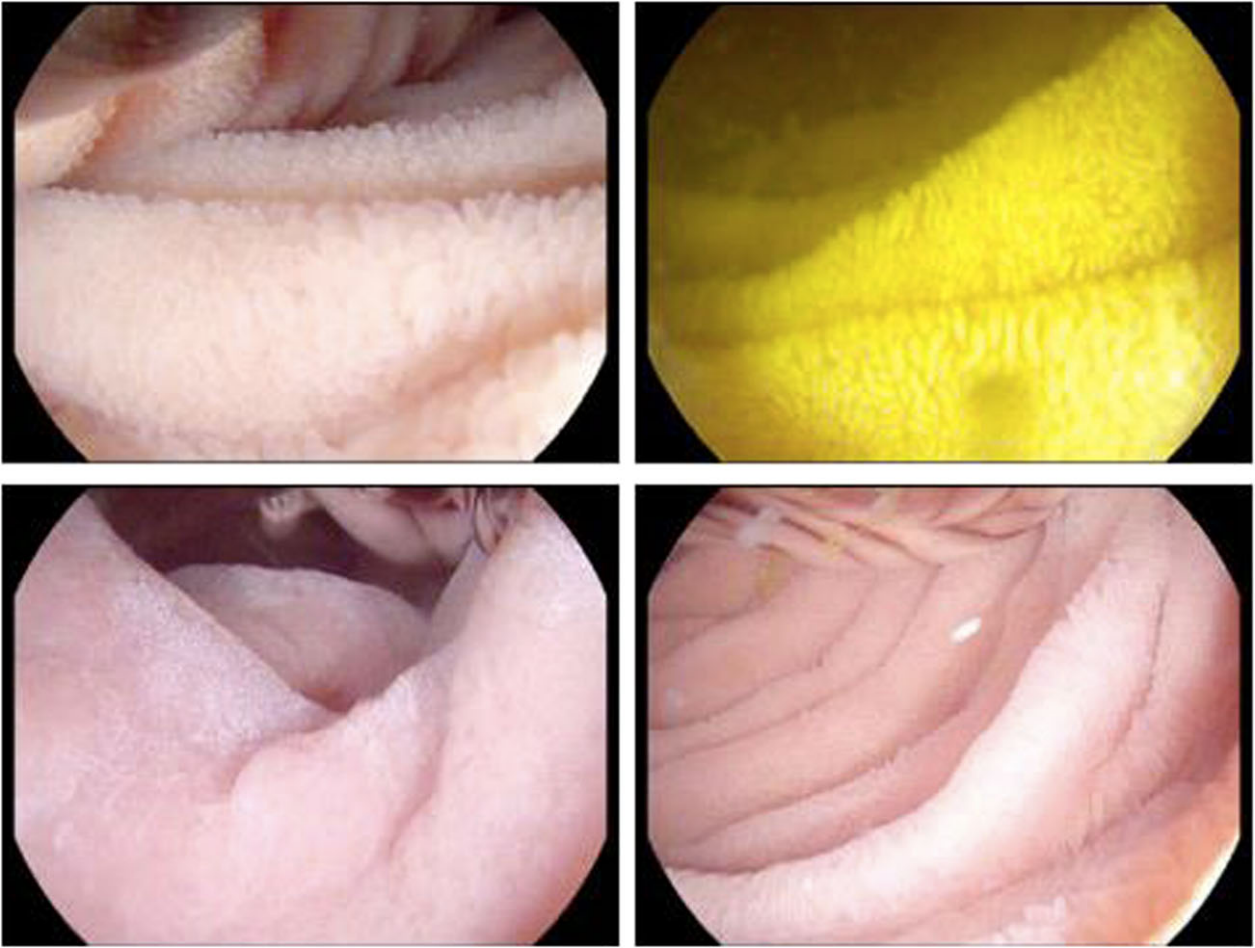
High-definition endoscopic images of small intestinal mucosa in EE, demonstrating increasingly severe enteropathic changes. *Clockwise from top left*: predominant leaves, predominant ridges, predominant convolutions, subtotal villous atrophy

The central feature of all the tropical enteropathies is villus remodelling, with blunting and reduction in surface area. This can be identified microscopically [12] (Fig. 1) or with high-definition endoscopes (Fig. 2).

Current thinking, however, is that in order to appreciate the full ramifications of enteropathy, particularly in order to measure long-term effects, it will be necessary to describe it in several domains or axes of measurement. These include villus blunting, mucosal inflammation, barrier failure, microbial translocation, systemic inflammation and malabsorption [19].

## Current Views About Environmental Enteropathy

### Causation

It is really not understood why the insanitary environment precipitates EE. Current hypotheses include:

- Environmental exposure to faecal organisms of human origin, including established enteropathogens [20, 21]
- Environmental exposure to faecal organisms of animal origin
- Mycotoxins [22]
- Nutrient deficiencies, including micronutrients and macronutrients
- Inflammation generated by other infectious processes such as recurrent respiratory infections [8]

There is no firm evidence for any of these. Two major trials of water and sanitation interventions are due to report main findings in 2017: the WASH-benefits trial (ISRCTNs: NCT01704105 and NCT01590095) and the SHINE trial (ISRCTN 14393738). These trials will test the faecal organisms hypotheses and, to a lesser extent, the nutrient deficiency hypotheses. Several micronutrient trials have failed to demonstrate benefit on EE [23], so if there is a nutrient deficiency underlying it, it has not yet been identified.

The complex reciprocal relationship between diet and mucosal inflammation has only recently been appreciated and remains poorly understood. This is particularly true of EE, where microbiological studies are only just beginning to be conducted. Bangladeshi children and adults living in an urban slum environment, where EE is highly prevalent, have significantly different microbiomes compared to healthy affluent US children [24]. In particular, Bangladeshi children and adults had enhanced levels of *Prevotella*, *Butyrivibrio* and *Oscillospira* and depleted in *Bacteroides* relative to US children; furthermore, the Bangladeshi microbiome was more variable over time when assessed on a monthly basis for up to 6 months [24]. Bangladeshi subjects had greatly reduced Bacteroidetes and greatly increased Firmicutes phyla, compared to US children. These children and adults remained healthy and diarrhoea- and helminth-free during the course of the study. Non-pathogenic microbes are frequently isolated from stool samples in individuals with EE. In one study in urban Zambia, for example, *Citrobacter rodentium* was overrepresented [12]. This bacterial species, although not known to be pathogenic in humans, is pro-inflammatory and induces ulcerative colitis in murine models and in cell culture systems. Populations where EE and stunting are prevalent tend to have a diet low in animal fat and protein and high in starch, fibre and plant polysaccharides. These dietary differences result in significant differences in the microbiome [25, 26•]. Comparing the faecal microbiome in children from rural Burkina Faso to Italy by 16S sequencing, for example, demonstrates a significant enrichment in Bacteroidetes and depletion in Firmicutes in Burkina Faso children [25]. This is of interest as pattern of phyla abundance is implicated in the pathogenesis of inflammatory bowel disease.

Animal models [27••, 28••] suggest that both infection and nutrient deficiency cooperate to produce enteropathy, but it is not yet clear that these truly reflect the human condition.

### Pathophysiology

The intestinal epithelium and its associated structures provide a physical barrier against the teeming luminal environment, while at the same time allowing adequate uptake and absorption of ingested nutrients and water [29, 30]. Impairment or dysregulation of barrier function results in both opportunity for the translocation of microbes and microbial products into the host [31] and loss of nutrients and water into the gut lumen. Barrier dysfunction may result from one or more of physical, microbiological or immunological insults [30]. EE is characterised by an impaired physical barrier between the intestinal lumen and the mucosal and submucosal vasculature, which is normally maintained by the epithelial barrier. This results in increased intestinal permeability, which results in loss of plasma constituents into the bowel lumen, and microbe and microbial product translocation. The normal physiological intestinal barrier comprises four interlinked components: the mucus barrier, the epithelium, the adaptive and innate contributions from the mucosal immune system [32, 33] and the microbiome. Intestinal mucus is composed of a variety of glycans secreted by epithelial goblet cells [34]. The mucus barrier provides a physical, biochemical and anti-microbial barrier to chemical, physical and microbiological insults. Secondly, it traps potentially harmful bacteria away from the intestinal epithelium. Furthermore, physiological mucus can promote the establishment and growth of beneficial/anti-inflammatory bacterial taxa (and conversely inhibits the growth of deleterious species) by providing them with taxa-specific nutrients. It is increasingly recognised that mucus composition and production are tightly regulated. Disorders of mucus production or composition predispose to intestinal inflammation and are observed in inflammatory bowel disease, but this has not been studied in EE.

**Fig. 3:**
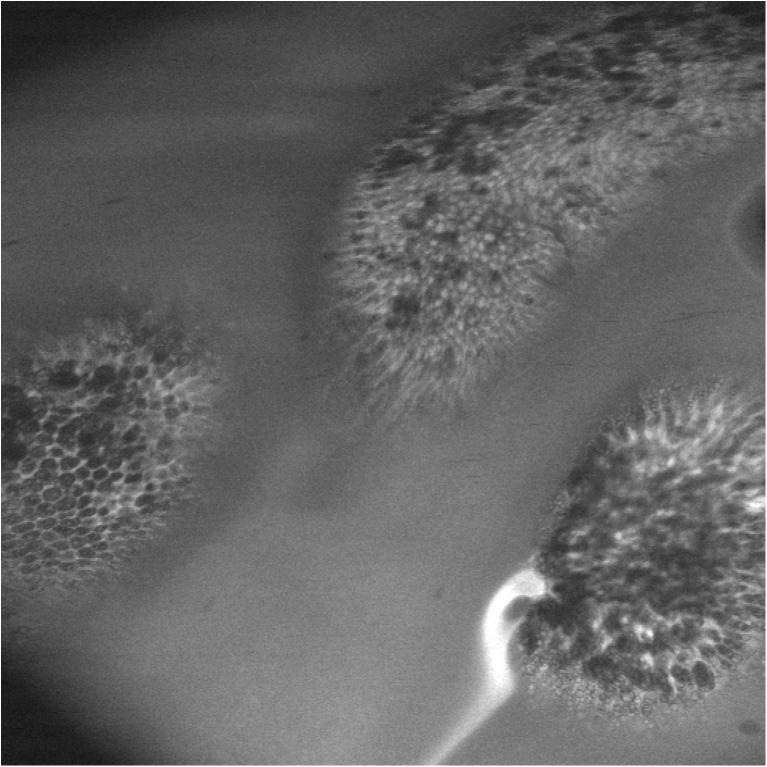
Confocal laser endomicroscopy of villus structures in environmental enteropathy in a Zambian adult. In contrast to the normal situation, in which fluorescein infected intravenously during the procedure is contained within the mucosa by tight junctions between epithelial cells, fluorescein here is observed leaking from the mucosa

Using confocal laser endomicroscopy, we have been able to identify epithelial lesions in vivo [35•]; prominent among which are microerosions and points of leakage which allow passive translocation of bacteria and bacterial components into the sub-epithelial compartment and transexudation of plasma constituents into the gut lumen (Fig. 3).

### Long-Term Sequelae of EE

Current thinking is that EE leads to stunting (failure of linear growth) [17••] and failure to respond to oral vaccines [36]. More speculatively, it has been suggested that other non-communicable diseases may originate in childhood enteropathies.

## Tropical Sprue

This is a very different disorder. The manifestations of this now-obscure disorder include persistent diarrhoea and profound weight loss. This is in total contradistinction to tropical/environmental enteropathy which by definition is completely asymptomatic. Tropical sprue has been reported only rarely from Africa [8], and most of these reports do not exclude other disorders, but was common in South Asia and the Caribbean. Tropical sprue has become rare, which might be explained if older tropical sprue cases were previously due to infections which can now be diagnosed with precision [37]. One of us (PK) has seen a case in East London in an immigrant from Bangladesh. The old definition of tropical sprue includes evidence of malabsorption of fat, vitamin B_12_ and xylose. This is difficult to establish in an age when clinical chemistry laboratories can no longer measure faecal fat or perform a Schilling test. We propose that in order to diagnose tropical sprue in the twenty-first century, it is necessary that the clinical picture is consistent with sprue (diarrhoea and weight loss), small intestinal biopsies are consistent with sprue (villus blunting, increased crypt depth and lymphocyte infiltration) and there is evidence of small intestinal malabsorption (low serum or red cell folate, a fat-soluble vitamin such as retinol) *in the absence of intestinal infection*.

## Malnutrition Enteropathy

Malnutrition underlies almost half of all child deaths globally and therefore contributes enormously to the unacceptably high under-5 mortality rates in these regions [38•]. Chronic undernutrition is usually manifest as stunting (poor linear growth), affects 30–40% of children in Zimbabwe and Zambia [39] and is associated with increased mortality [40•], reduced neurodevelopmental potential and decreased long-term economic productivity [41]. Current thinking is that environmental enteropathy makes a major contribution to chronic undernutrition [17••]. Acute malnutrition is usually manifest as wasting (loss of tissue), the most conspicuous of all nutritional disorders. Severe acute malnutrition (SAM) carries the highest mortality [42], particularly if associated with complications. Children with clinical complications of SAM requiring hospital treatment often fail to respond to treatment [43] and continue to experience high mortality of up to 35% [42, 44]. Even after discharge, children have a poor prognosis, with 42% mortality over the subsequent year [45]. In our experience, it is a subgroup of children with SAM and persistent diarrhoea who pose the most difficult management challenges, although the vast majority of children with SAM have a degree of enteropathy [44, 46]. Indeed, the intimate relationship between malnutrition and a ‘chronic derangement of the alimentary canal’ was described among the British poor in 1868 [47]. A high pathogen burden causes damage to the mucosa which exacerbates nutritional impairment and leads to further susceptibility to infection and impaired epithelial regeneration in a cyclical process first described in Central America in the 1970s [48, 49]. This mucosal damage in SAM we here refer to as *malnutrition enteropathy*, and it is associated with very dramatic elevations of inflammatory molecules in the blood. The sequence of events, however, is not understood. Clinical experience among physicians who treat malnutrition suggests that children with moderate acute malnutrition (MAM) often progress to SAM, but it is not clear if it is nutrient deprivation per se which drives this or if it is infectious or inflammatory processes which drive progression. SAM may be complicated by oedema (‘kwashiorkor’), and we have some evidence that this is associated with glycosylation defects [50].

It may be instructive to contrast two radically different extreme malnutrition disorders. In victims of the Nazi genocides of the 1940s, extreme malnutrition and intestinal atrophy were characterised by universal and often fatal diarrhoea [51, 52], and one Hungarian physician postulated that extreme malnutrition ‘rendered the intestines extraordinarily susceptible to infection [53]. Many physicians working in the appalling conditions of the concentrations camps expressed the view that malnutrition diarrhoea is not infectious in aetiology [51, 54]. There are data from animal models which suggest that pure starvation can induce a hypersecretory state in the small intestine [55–57]. In contrast, extreme malnutrition in patients with anorexia nervosa is not associated with diarrhoea [58] despite what must inevitably be a very advanced state of intestinal atrophy. For obvious reasons, it is difficult to study intestinal structure and function in the circumstances in which extreme malnutrition occurs, so our understanding of enteropathy in severe malnutrition is rudimentary and much work is needed to understand how to reverse the enteropathy.

## Contrasting Tropical with Other Enteropathies

The spectrum of enteropathies is increasing, though a detailed description is beyond the scope of this review. Coeliac disease is prevalent all over the globe, though our experience is that it is uncommon in Zambia where we have most experience. Autoimmune enteropathy is rare everywhere and characterised by autoantibodies. NSAID-induced enteropathy is well described [59]. Recently, an olmesartan-related enteropathy has been described [60], and the genetic basis for an enteropathy related to defective prostaglandin transport has been reported [61]. Although a number of enteropathies look superficially similar to EE, there are recognisable differences. Where there are sufficient data to comment, these enteropathies are contrasted in Table 1. There are marked differences in responses to treatment: children with kwashiorkor and marasmus—both forms of severe acute malnutrition—respond to nutritional treatment, and we assume that this includes the enteropathy of malnutrition. Tropical sprue responds to tetracycline and folic acid. However, the other enteropathies do not respond to either.

**Table 1:** Similarities and differences between various small bowel enteropathies

Condition	Distribution	Mucosal inflammation	Barrier defect	Glycosylation defect	Systemic inflammation	Diarrhoea	Malabsorption	Weight loss	Response to antibiotics	Response to nutritional therapy	Mortality
Environmental enteropathy	Proximal	+	+	?	+	−	Subclinical only	−	−	−	−
Kwashiorkor	Not defined	++	++	++	++	+/−	+	++	+	+	++
Marasmus	Not defined	++	++	−	++	+/−	+	++	+	+	++
Coeliac disease	Proximal	+	+	?	−	+	+	+	−	−	Rare
GVHD	Variable	+	+	?	−	+	Probably	+	−	−	++
Tropical sprue	Global, often distal	+	+	?	?	++	++	++	+	+	Rare
Autoimmune enteropathy	Global	++	?	?	?	++	++	+	−	−	++
Olmesartan enteropathy	Global	++	?	?	?	+	+	+	−	−	−

Kwashiorkor is a term used to describe severe acute malnutrition with oedema, and marasmus to describe severe acute malnutrition without oedema

*GvHD* graft-versus-host disease

## Conclusions

There are several enteropathies which can occur in individuals living in tropical regions, but only tropical sprue is exclusive to tropical populations. Tropical sprue is likely to be purely infectious, but environmental enteropathy is probably due to a combination of infectious and nutritional derangements. Whether malnutrition enteropathy is a distinct disorder due to severe nutritional depletion, or merely the severe end of a spectrum of enteropathies due to interactions between infection and nutrient depletion, is not clear at this time. Any other enteropathic disorder may be seen in a patient in a tropical region, just as they may be in any population centre. More work is needed to determine how best to heal the mucosal defect in children with environmental enteropathy or severe acute malnutrition, as it is the children who bear the most severe consequences of these disorders in the tropics.
